# Movement Disorders in Childhood Thalamic Tumors

**DOI:** 10.15844/pedneurbriefs-32-4

**Published:** 2018-08-28

**Authors:** Rebecca Garcia-Sosa, Joanna Blackburn

**Affiliations:** 1 Division of Neurology, Ann & Robert H Lurie Children’s Hospital of Chicago, Chicago, IL; 2 Departments of Pediatrics and Neurology, Northwestern University Feinberg School of Medicine, Chicago, IL

**Keywords:** Movement Disorders, Thalamic Tumors, Low Grade Gliomas, Red Nucleus

## Abstract

Investigators from St. Jude Children’s Research Hospital in Memphis, Tennessee analyzed movement disorder outcomes in patients with childhood thalamic tumors.

Investigators from St. Jude Children’s Research Hospital in Memphis, Tennessee analyzed movement disorder outcomes in patients with childhood thalamic tumors. Most of the thalamic tumors were low grade gliomas. The tumors themselves and the surgical interventions required for their treatment can result in neurological complications including motor and/or sensory deficits, visual deficits, seizures, alterations in intracranial pressure and movement disorders. They retrospectively reviewed the charts of 83 patients treated at their institution over 17 years to further evaluate the incidence, types and severity of hyperkinetic and/or hypokinetic movement disorders in this population. Nine out of the 83 patients studied (11%) developed one or more movement disorders. Most movement disorders began shortly after surgical resection and only one developed symptoms months prior to surgery. The most common movement disorder was postural tremor, followed by ballism, athetosis, resting tremor, myoclonus and dystonia. Study neurologists were blinded to magnetic resonance imaging (MRI) data in order to analyze the extent of involvement of the thalamic nuclei. Out of the eight thalamic nuclei identified as affected in the MRI data of these patients, only the red nucleus was involved in all nine patients. Six of these patients also had substantia nigra involvement. Outcomes were assessed using the Karknofsky Performance Scale (KPS), the Extrapyramidal Symptoms Rating Scale (ESRS), and the Clinical Global Impression of Severity (CGI-S) with a median follow up of three years. Severity of the movement disorder remained unchanged to slightly improved (4/9) in follow up despite several medication trials in all but one patient (8/9). [1]

COMMENTARY. This article finds that movement disorders associated with thalamic tumors are more commonly seen as a sequelae of surgical injury than as a presenting symptom of the tumor. Although the findings in this article focus on the occurence and outcome of movement disorders that develop after surgical intervention it is important to remember that brain neoplasms and other focal lesions need to be considered when otherwise healthy children develop the sub-acute onset of bilateral or unilateral tremor as well as other types of movement disorders [2]. While movement disorders associated with brain tumors are more commonly seen unilaterally, it is important to note that in this study, a subset of patients developed bilateral symptoms. Another important finding in this study is that the movement disorders did not improve significantly over time. This suggests a permanent injury from the tumor or surgical procedure, rather than post- operative inflammation, as the cause of the movement disorder associated with thalamic tumors. Whether a movement disorder develops before or after the diagnosis and treatment of a thalamic tumor, they represent significant morbidity for this population and can have a great impact in their quality of life. When addressing movement disorders associated with thalamic tumors, it is imperative to identify the correct phenomenology in order to implement appropriate pharmacological and non-pharmacological interventions that are appropriate for each individual patient [3]. Even with correct phenomenology identification, secondary movement disorders are difficult to treat regardless of the underlying cause of the involuntary movement. In these settings, it is common for multiple medications to be tried without success. As investigators suggested, the treatment of secondary movements disorders merits further research. Treatment can be extremely challenging and many times ineffective in this this vulnerable patient population.

## Disclosures

The author(s) have declared that no competing interests exist.
